# Rosazea im Kindes‐ und Jugendalter

**DOI:** 10.1111/ddg.15693_g

**Published:** 2025-06-11

**Authors:** Sören Korsing, Karola Stieler, Uwe Pleyer, Ulrike Blume‐Peytavi, Annika Vogt

**Affiliations:** ^1^ Klinik für Dermatologie Venerologie und Allergologie Charité – Universitätsmedizin Berlin; ^2^ Klinik für Augenheilkunde Charité – Universitätsmedizin Berlin; ^3^ Berlin Institute of Health Charité – Universitätsmedizin Berlin

**Keywords:** Demodex, IFAG, Kinderdermatologie, Rosazea, Demodex, IFAG, Pediatric Dermatology, Rosacea

## Abstract

Obwohl die Rosazea bei Kindern und Jugendlichen ähnliche Symptome, Auslöser und Verlaufsmuster wie bei Erwachsenen zeigt, wird sie in diesen Altersgruppen als Differenzialdiagnose häufig erst nachrangig in Betracht gezogen. Erstmanifestationen klassischer klinischer Typen können jedoch bereits ab dem Kleinkindalter beobachtet werden. Sonderformen wie das idiopathische aseptische faziale Granulom sowie die häufige okuläre Beteiligung stellen Besonderheiten der kindlichen Rosazea dar.

Therapeutische Optionen für Rosazea im Kindes‐ und Jugendalter überlappen sich teilweise mit dem Vorgehen bei häufigen Differenzialdiagnosen wie der Acne vulgaris oder der perioralen Dermatitis. Allerdings bringen sowohl die Erkrankung selbst als auch das Auftreten auf kindlicher Haut Besonderheiten bei der Auswahl der Wirkstoffe und der Erstellung der Therapiekonzepte mit sich. Zudem bedarf es einer Beratung zu Trigger‐ und Aggravationsfaktoren, um die Betroffenen auch langfristig bestmöglich zu unterstützen.

## EPIDEMIOLOGIE UND PRÄDISPONIERENDE FAKTOREN

Die Rosazea ist eine bei Erwachsenen häufig zwischen dem 30.–50. Lebensjahr auftretende, chronisch‐entzündliche Erkrankung, die vor allem die zentrale Gesichtshaut befällt. Bei Kindern und Jugendlichen wird die Rosazea seltener in Betracht gezogen, jedoch vermutlich unter‐ oder fehldiagnostiziert.^1^ Dies ist möglicherweise durch das geringere Bewusstsein über die Erkrankung in dieser Altersgruppe, morphologische Ähnlichkeiten mit der viel häufigeren Acne vulgaris oder auch klinische Unterschiede im Vergleich zum Erwachsenenalter (zum Beispiel idiopathisches faziales aseptisches Granulom oder periorifizielle Variante) bedingt.

Genaue Daten zur Inzidenz und Prävalenz existieren nicht. Die Erstmanifestation erfolgt meist zwischen dem 4. und 8. Lebensjahr. Bis zur Diagnosestellung vergehen oft mehrere Monate bis Jahre. Die prädisponierenden Faktoren scheinen denen der erwachsenen Patienten zu entsprechen: heller photobiologischer Hauttyp I–II nach Fitzpatrick, insbesondere bei papulopustulöser Variante, sowie die Anwendung topischer oder inhalativer Steroide, insbesondere bei periorifizieller Variante.^2^, ^3^ Ein Auftreten bei Kindern und Jugendlichen dunklerer Hauttypen ist möglich.^4^, ^5^ Ob bei dunkleren Hauttypen jedoch wirklich eine geringere Inzidenz oder hier eine diagnostische Lücke besteht, ist bislang nicht geklärt.^6^

Bezüglich der Geschlechtspräferenz liegt eine uneinheitliche Datenlage vor, die meist eine ausgeglichene Verteilung aufzeigt.^7^ Aus wenigen Arbeiten geht ein häufigeres Auftreten bei weiblichen Patientinnen hervor, insbesondere bei Ophthalmorosazea mit schwerem Verlauf.^2^

## PATHOGENESE

Die Pathogenese der Rosazea im Kindes‐ und Jugendalter unterscheidet sich nach aktuellem Wissensstand nicht von der bei Erwachsenen und basiert auf prädisponierenden, genetischen Faktoren und externen Triggerfaktoren. Eine Dysregulation des angeborenen und erworbenen Immunsystems, eine vasomotorische Instabilität sowie die Interaktion mit Mikroorganismen der Haut sind pathogenetisch relevant.^8^ Als Triggerfaktoren für die Manifestation einer Rosazea werden altersunabhängig ultraviolette Strahlung, Hitze, scharfe oder heiße Nahrungsmittel und Getränke, topische Steroide und psychische Belastung gesehen.^9^

Wie bei Erwachsenen kann bei pädiatrischen Patienten eine familiäre Häufung der Rosazea vorliegen.^10^ Einzelne Kandidaten‐Gene und zwei Einzelnukleotid‐Polymorphismen, die mit der Rosazea assoziiert sind, wurden identifiziert.^11^ Das Vorliegen einer Gain‐of‐Function‐Mutation im *STAT1*‐Gen resultiert in einem Immundefekt mit assoziierter Überbesiedlung von Demodex‐Milben. Dermatologisches Leitsymptom bei diesem Immundefekt kann das frühe Auftreten Rosazea‐artiger Veränderungen sein.^12^, ^13^

Auf molekularer Ebene kommt Cathelicidinen eine zentrale Rolle zu. Diese antimikrobiellen Peptide werden unter anderem durch Mastzellen freigesetzt, induzieren proinflammatorische Botenstoffe wie Interleukin (IL)‐8 und fördern die Neovaskularisation über die Stimulation von *vascular endothelial growth factor* (VEGF). Das vermehrte Vorkommen von Cathelicidinen in der Haut von Patienten mit Rosazea ist auf die erhöhte Produktion von Vorläufermolekülen sowie eine erhöhte Expression von Matrix‐Metalloproteasen wie Kallikrein zurückzuführen.^14^, ^15^ Cathelicidine werden über den Toll‐like‐Rezeptor 2 (TLR2) induziert, der bei Rosazea überexprimiert wird.^16^ Mikroorganismen wie *Staphylococcus epidermidis* und Demodex‐Milben sind über die Aktivierung von TLR2 an der Initiierung einer Immunantwort bei der Rosazea beteiligt.^17^ Immunkompetente Kinder mit gesunder Haut sind in der Regel nicht von Demodex‐Milben befallen. Die Besiedelung nimmt während des Heranwachsens zu.^18^ Erfolgreiche Therapieversuche, die auf einer Reduktion der Demodex‐Besiedelung bei Kindern mit Rosazea basieren, lassen auch in dieser Altersgruppe auf einen pathophysiologischen Zusammenhang schließen.^19^ Der Nachweis von Demodex‐Milben ist bei Rosazea‐Patienten jedoch nicht obligat.

Auch das adaptive Immunsystem ist in Form einer durch Typ‐1‐T‐Helferzellen dominierten Immunantwort in die Pathogenese der Rosazea involviert. Diese zieht die Hochregulation weiterer proinflammatorischer Zytokine wie Tumornekrosefaktor (TNF)‐α und IL‐17 nach sich.^20^

Schließlich spielt eine neurovaskuläre Dysregulation eine relevante Rolle. Bei Personen mit entsprechender Prädisposition führen die typischen Triggerfaktoren der Rosazea zu einer erhöhten Aktivierung von Transient‐Receptor‐Potential‐Vanilloid‐4 (TRPV4)‐Ionenkanälen.^21^

## DIAGNOSESTELLUNG UND KLINISCHE PRÄSENTATION

Die Diagnosestellung der Rosazea erfolgt auf der Grundlage des klinischen Befundes. Dieser kann sich bei pädiatrischen Patienten sehr ähnlich zur Erkrankung des Erwachsenenaltes darstellen.

**ABBILDUNG 1:**
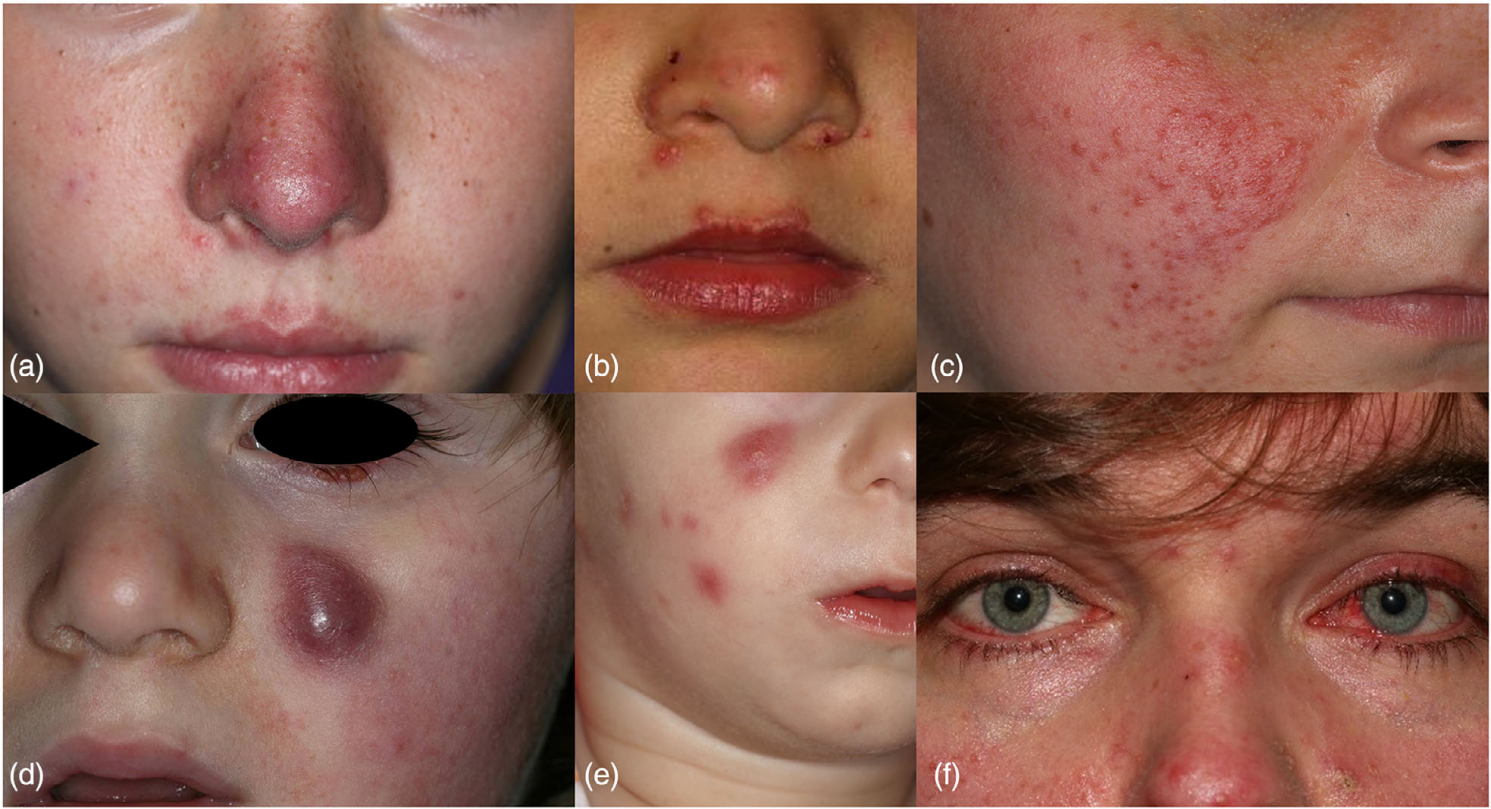
Klinische Phänotypen der Rosazea im Kindes‐ und Jugendalter: (a) Kombinierte erythematöse Rosazea (ER) und papulopustulöse Rosazea (PPR), (b) Periorifizielle Rosazea (POR), (c) granulomatöse Rosazea (GR), idiopathisches faziales aseptisches Granulom (IFAG) mit (d) solitärer Läsion und Wangenerythem sowie (e) multiplen Läsionen, (f) Ophthalmorosacea (OR).

Während epidemiologische Daten darauf hindeuten, dass 57% der Rosazea‐Fälle aller Altersgruppen dem erythematösem beziehungsweise teleangiektatischen, 43% dem papulopustulösen, 7% dem phymatösen und 11% dem okulären Subtyp angehören, fehlen genaue Daten für pädiatrische Patienten.^6^ Aus veröffentlichten Fallserien bei Kindern wird jedoch deutlich, dass periorifizielle und okuläre Manifestationen sowie schwere Formen der idiopathischen fazialen aseptischen Granulome im Gegensatz zu den häufigen erythematös‐teleangiektatischen und papulopustulösen Subtypen klinische Herausforderungen darstellen (Tabelle 1, Abbildung 1).^22^ Die phymatöse Form wurde in der jungen Altersgruppe bislang nicht beschrieben.^23^

**TABELLE 1:** Subtyp und Charakteristika der Rosazea im Kindes‐ und Jugendalter.

**Subtyp**	**Charakteristika**
Erythematöse beziehungsweise teleangiektatische Rosazea (ER)	Faziale Flushs und/oder persistierende Erytheme
Papulopustulöse Rosazea (PPR)	Papeln und Pusteln konvexer Gesichtsareale (Wangen, Stirn, Kinn)
Periorifizielle Variante (POR)^*^	Periorifizielle Lokalisation der Papulopusteln, meist perioral mit Beteiligung des Philtrums
Granulomatöse Variante (GR)	Derbe Papeln und Nodi, granulomatöses Infiltrat
Idiopathisches faziales aseptisches Granulom (IFAG)^*^	Solitäre oder multiple erythematöse bis livide Nodi (oft infraorbital/ an den Wangen)
Ophthalmorosacea (OR)^*^	Blepharitiden, Konjunktivitiden, Chalazia, Hordeola oder korneale Affektionen (Keratitiden, Neovaskularisationen, Ulzera)

* Obwohl genaue Daten über die Verteilung der Subtypen bei Kindern und Jugendlichen fehlen, zeigen eigene Erfahrungen und die Darstellung in Fallserien und Therapieberichten, dass die hervorgehobenen Subtypen rezidivierende und manchmal therapeutisch schwer erreichbare Entitäten in diesen Altersgruppen darstellen.

Bislang wurden keine international anerkannten Diagnosekriterien der Rosazea im Kindes‐ und Jugendalter definiert.^24^ Wir verweisen bezüglich der Diagnosestellung jedoch aufgrund der Überschneidungen zum Erwachsenenalter auf die aktuelle S2k‐Leitlinie.^9^ Chamaillard et al. schlagen die folgenden Kriterien bei pädiatrischen Patienten vor, wobei zwei Kriterien zur Diagnosestellung vorliegen müssen:

‐ Flushs oder persistierende Gesichtserytheme,
‐ faziale Teleangiektasien, die nicht durch andere Ursachen erklärt werden können,
‐ papulopustulöse Läsionen, Fehlen von Komedonen,
‐ Lokalisation der Läsionen auf konvexen Arealen des Gesichts,
‐ okuläre Beteiligung (Blepharitis, rezidivierende Chalazia, Konjunktivitis, Keratitis).^7^

Zusätzlich können subjektive Symptome wie Pruritus, Brennen oder Stechen und ein Trockenheitsgefühl auftreten.^9^ Biopsien oder andere weiterführende Diagnostik sind in der Regel nicht erforderlich und dienen bei unklaren Fällen der Abklärung anderer Differenzialdiagnosen. Es existieren wenige Fallberichte über die histopathologischen Charakteristika pädiatrischer Patienten mit Rosazea. Diese scheinen sich aber nicht von denen der Erwachsenen zu unterscheiden.^25^ Spezielle Tape‐Abrissmethoden, unter Einsatz eines Cyanoacrylat‐Klebstoffs, können in einigen Fällen den Nachweis einer erhöhten Demodex‐Besiedlung erbringen, sollten angesichts der empfindlichen Haut der Patienten allerdings besonderen Fällen vorbehalten bleiben.^26^ Da das Erscheinungsbild je nach klinischem Typ variiert, ergibt sich je nach Ausprägung ein unterschiedliches Spektrum an möglichen Differenzialdiagnosen.

### Erythematöse beziehungsweise teleangiektatische Rosazea (ER)

Die Flushs verbleiben länger als wenige Minuten oder persistieren und unterscheiden sich somit von physiologischen Erythemen. Sie betreffen insbesondere die Wangenregion. Flushs können durch Hitze oder UV‐Licht getriggert werden. Makroskopisch sichtbare Teleangiektasien können auftreten.^1^, ^3^, ^7^ Das klinische Bild der ER kann der Entwicklung papulopustulöser Läsionen vorausgehen.^10^

### Papulopustulöse Rosazea (PPR)

Die papulopustulöse Rosazea ist die am häufigsten beschriebene Variante im Kindes‐ und Jugendalter. Überschneidungen mit der ER und POR sind möglich (Abbildung 1a). Subjektive Symptome wie Pruritus, Dysästhesien oder Hautbrennen werden selten beschrieben.^1^, ^7^ Eine Akne vulgaris kann zeitgleich vorliegen.

Eine Induktion oder Exazerbation durch Steroide ist möglich (Steroid‐Rosazea). Die Rosazea fulminans der Erwachsenen ist als seltene, akut verlaufende Maximalvariante der papulopustulösen Rosazea definiert, durch Knoten, Abszesse und Fisteln gekennzeichnet und insbesondere bei schwangeren Frauen beschrieben. Es existieren wenige Fallberichte über die Rosazea fulminans bei Jugendlichen sowie ein Fallbericht über eine 3‐jährige Patientin.^27^, ^28^

### Periorifizielle Rosazea (POR)

Die papulopustulösen Läsionen zeigen eine meist periorale Verteilung mit Beteiligung des Philtrums (Abbildung 1b). Weitere Varianten sind perinasal oder periokulär lokalisiert. Eine Abgrenzung zur klassischen perioralen Dermatitis, die durch zu reichhaltige Pflege und topische Steroide induziert wird, ist nicht möglich. Einige Autoren postulieren, dass die periorale Dermatitis bei Kindern und Jugendlichen stets als Rosazea einzuordnen ist. Hierfür spricht, dass eine klinische und histopathologische Differenzierung nicht möglich ist und die Läsionen auf die gleichen Therapieprinzipien ansprechen.^29^, ^30^

### Granulomatöse Rosazea (GR)

Die Variante ist äußerst selten beschrieben und stellt sich klinisch durch Follikel‐assoziierte, braunrote Knötchen dar (Abbildung 1c). Unter Glasspatel‐Druck kann ein Apfelgelee‐artiges Infiltrat imponieren. Histopathologisch zeigen sich Granulome.^31^

### Idiopathisches faziales aseptisches Granulom (IFAG)

Das idiopathische faziale aseptische Granulom wird als Variante der GR betrachtet und manifestiert sich im Kleinkindalter durch solitäre (Abbildung 1d) oder selten multiple (Abbildung 1e), erythematöse Knoten, die typischerweise infraorbital oder an den Wangen lokalisiert sind. Periphere Entzündungszeichen fehlen. Ein subjektiver Leidensdruck besteht in der Regel nicht. Kinder stellen sich oftmals nach operativer Inzision oder bioptischer Sicherung vor.^32^, ^33^

Die Sonographie kann für Diagnose und Verlaufsbeurteilung hilfreich sein.^34^ Histologisch zeigen sich in variabler Ausprägung teils lymphohistiozytäre Infiltrate, Follikulitis oder Granulome. In der Regel kommt es nach circa 1 Jahr zur Spontanregredienz ohne Narbenbildung oder Rezidive, sodass ein abwartendes Verhalten erwogen werden kann. Alternativ zeigt sich ein variables Ansprechen unter topischen oder systemischen Antibiotika sowie Isotretinoin in schweren Fällen.^35^ Betroffene Kinder können weitere klinische Rosazea‐Zeichen nach Chamaillard et al. zeigen.^7^, ^35^ Bei Erwachsenen tritt dieser Phänotyp nicht auf. Teilweise kann die Abgrenzung von entzündeten Zysten, oder auch tumorösen Prozessen wie Pilomatrikomen nicht einfach sein. Daher ist das Wissen um diese Entität hilfreich, um unnötige chirurgische Interventionen zu vermeiden.

### Ophthalmorosacea (OR)

**ABBILDUNG 2:**
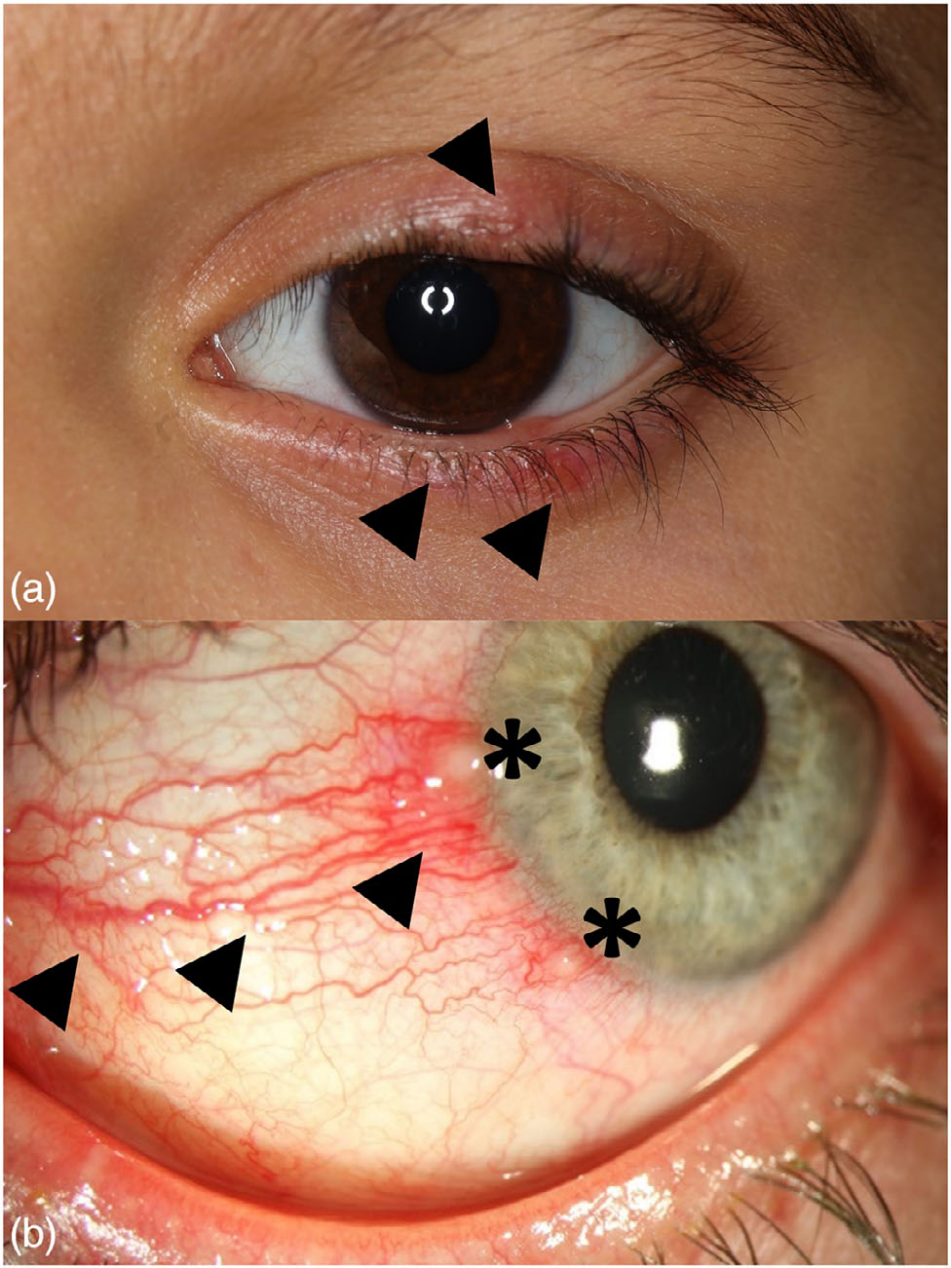
Befund bei Ophthalmorosacea (OR): (a) Blepharitis mit Liderythem und Papulovesikeln des Unterlids (Pfeil), (b) Schwere Blepharokonjunktivitis mit ausgeprägten Neovaskularisationen (Pfeil) und Ulzerationen (Stern)

Okuläre Läsionen treten bei bis zu 60% der Kinder und Jugendlichen und damit häufiger als bei Erwachsenen auf.^2^, ^36^, ^37^ Die OR ist häufig bilateral lokalisiert (Abbildungen 1, 2).^38^ Weibliche Kinder und Jugendliche scheinen häufiger und schwerer zu erkranken und berichten über Trockenheits‐ und Fremdkörpergefühl, Brennen und Tränenbildung, Photosensitivität, Rötungen der Konjunktiven und Augenlider oder Schwellungen der Orbital‐ und Periorbitalregion.^2^ Bei etwa der Hälfte der jungen Patienten gehen die okulären Symptome und Befunde den kutanen Zeichen voraus.^7^, ^39^ Zudem können diese isoliert auftreten, was die Diagnosestellung erschwert. Liegt eine kombinierte okulokutane Rosazea vor, besteht oftmals ein papulopustulöser Phänotyp.^40^

Als initiale klinische Veränderung dominiert eine Meibom‐Drüsen‐Dysfunktion mit übergreifender Entzündung auf die Augenoberfläche (Konjunktivitis, Keratitis). Bei Spaltlampenuntersuchung der Lidränder zeigen sich Teleangiektasien, erweiterte Meibom‐Drüsen mit Sekretverhalt und „Kollaretten“. Aus der Meibom‐Drüsen‐Dysfunktion resultieren Störungen der Lipidkomposition des Tränenfilms mit erhöhter Tränenverdunstung, verminderter Tränenfilm‐Aufrisszeit und ein evaporatives, trockenes Auge. Nicht selten können rezidivierende Entzündungen und Rötungen am Lidrand einen wichtigen zusätzlichen diagnostischen Hinweis zur Abgrenzung von einer Acne vulgaris liefern.^41^

### Morbus Morbihan

**ABBILDUNG 3:**
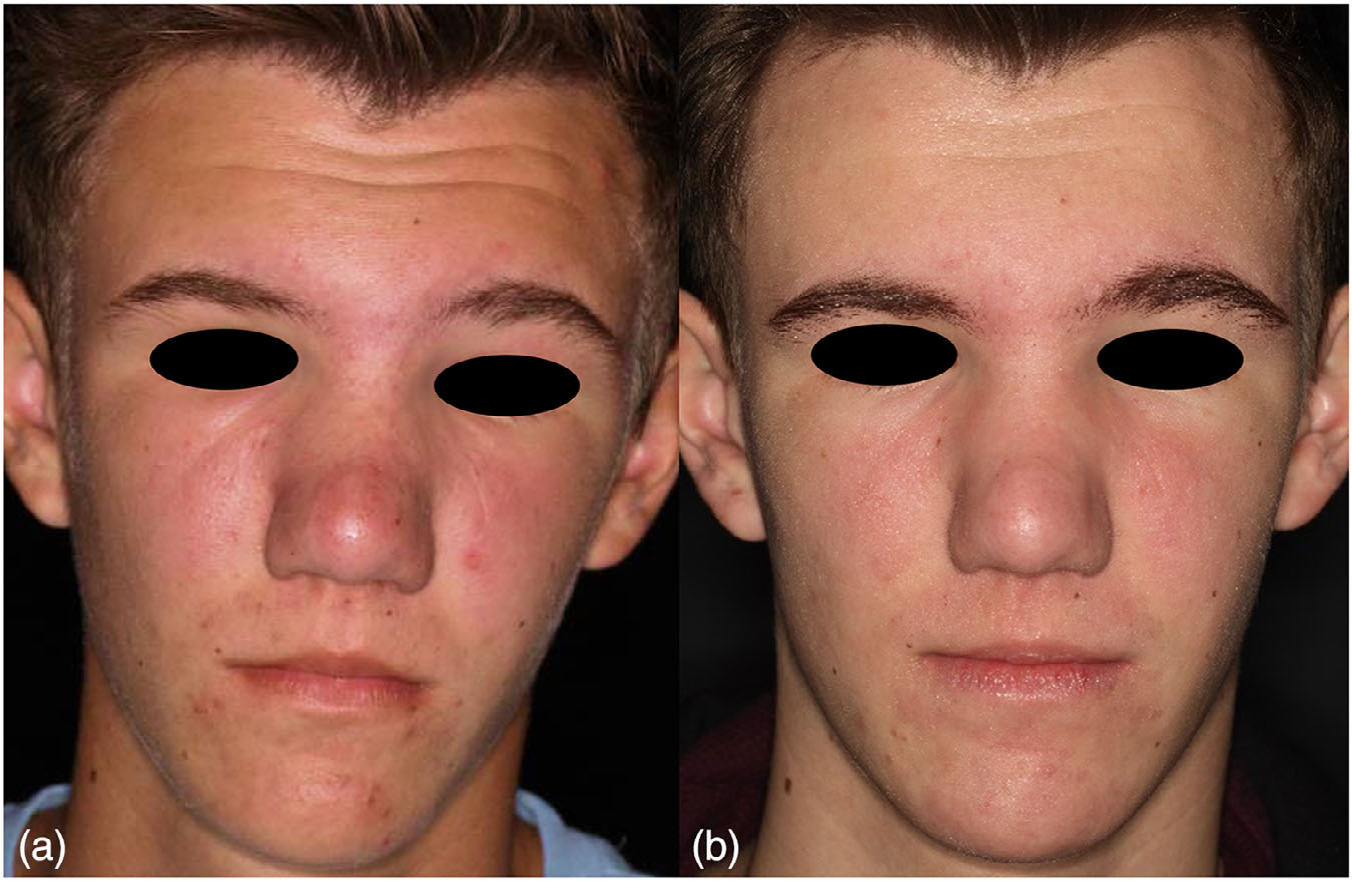
Morbus Morbihan: (a) 15‐jähriger Jugendlicher mit papulopustulösen Läsionen der Wangen und Perioralregion, derben, polsterartigen Schwellungen der Wangen, Nase und Glabellaregion mit subjektivem starken Spannungsgefühl. (b) Deutliche Besserung der Papulopusteln, des Erythems und Abnahme der Schwellungen nach dreimonatiger Systemtherapie mit Isotretinoin und manueller Lymphdrainage.

Hierbei handelt es sich um eine Sondervariante der Rosazea, die sich klinisch durch persistierende, Polster‐artige Schwellungen konvexer und periokulärer Gesichtsareale mit oder ohne weitere entzündliche Effloreszenzen präsentiert. Männliche Individuen sind häufiger betroffen.^6^, ^42^ Nach unserem Kenntnisstand existieren keine Fallberichtete zum Morbus Morbihan beziehungsweise fazialen Lymphödemen im Zusammenhang zur Rosazea pädiatrischer Patienten. Unserer klinischen Erfahrung nach können jedoch, wenn auch selten, ebenso in dieser Patientengruppe moderate Lymphödeme beobachtet werden (Abbildung 3).

## DIFFERENZIALDIAGNOSEN

Die Rosazea im Kindes‐ und Jugendalter tritt selten auf, daher muss je nach klinischer Manifestation ein breites Spektrum an Differenzialdiagnosen ausgeschlossen werden.

Da das IFAG typischerweise uniläsional bei Kleinkindern auftritt, gehören zu den wichtigsten Differenzialdiagnosen Pilomatrixome, juvenile Xanthogranulome und der Spitz Nävus. Weitere Differenzialdiagnosen sind Epidermalzysten, Chalazia, infektiöse Dermatosen (zum Beispiel Mykobakteriose und kutane Leishmaniose) und Fremdkörpergranulome.^3^

Das Gianotti‐Crosti‐Syndrom und die benigne zephalische Histiozytose sollten bei Säuglingen und Kleinkindern in Betracht gezogen werden, wenn disseminierte Gesichtsläsionen vorhanden sind. Bei persistierendem Gesichtserythem sollte an ein Schmetterlingserythem bei systemischem Lupus erythematodes oder auch an ein Ulerythema ophryogenes gedacht werden.

Bei Jugendlichen ist die Acne vulgaris eine wichtige Differenzialdiagnose für papulopustulöse Läsionen, die durch das Vorhandensein offener oder geschlossener Komedonen gekennzeichnet ist. Faziale Flushs, persistierendes Gesichtserythem und Teleangiektasien fehlen bei der Akne.

Weitere Differenzialdiagnosen in allen Altersgruppen umfassen die irritativ‐toxische und allergische Kontaktdermatitis, die seborrhoische Dermatitis, eine Demodikose (immunsupprimierte Patienten), die Sarkoidose und die Granulosis rubra nasi.

Bei okulärer Rosazea sollte differenzialdiagnostisch an virale oder bakterielle Infektionen, irritativ‐toxische oder allergische Ursachen oder eine atopische Dermatitis gedacht werden.

## THERAPIE

Die Behandlung der Rosazea bei Kindern und Jugendlichen basiert weitgehend auf den Therapiestrategien für Erwachsene, wobei die besonderen Anforderungen der jungen Patienten berücksichtigt werden müssen. Sowohl bei Kindern als auch bei Erwachsenen zielt die Rosazea‐Behandlung auf die Linderung der subjektiven Symptome, die Reduktion der entzündlichen Läsionen sowie die Verbesserung der Lebensqualität ab und wird je nach klinischem Phänotyp und Schweregrad modifiziert. Im Wesentlichen können die Empfehlungen der aktuellen S2k‐Leitlinie wiedergegeben werden, in welcher auch auf das Auftreten in jungem Alter verwiesen wird.^9^ Eine besondere Herausforderung stellt allerdings die Tatsache dar, dass alle Therapeutika im Off‐Label‐Use (OLU) angewendet werden, da diese entweder erst im Erwachsenenalter oder nicht primär für die Rosazea zugelassen sind. Zusätzlich müssen Altersgrenzen bei der Auswahl der Therapeutika bedacht werden, zum Beispiel beim Einsatz von Tetrazyklinen.

Basismaßnahmen umfassen die Identifikation und Vermeidung von Triggerfaktoren wie Hitze, UV‐Licht oder irritative Externa sowie Steroide. Weiterhin ist die Anwendung milder Reinigungsmittel und pflegender Externa zu empfehlen. Tagsüber sind UV‐Schutz‐Maßnahmen anzuwenden.^9^

Topische Therapiemaßnahmen werden bei leichtem bis moderatem Schweregrad eingesetzt, während Systemtherapien in Kombination mit topischen Maßnahmen bei hohem Schweregrad zum Einsatz kommen. Für die eingesetzten Therapeutika bei pädiatrischen Patienten existiert eine nur spärliche Datenlage, die sich hauptsächlich auf Einzelfallberichte, Fallserien und retrospektive Datenauswertungen stützt.^22^, ^43^ Die Tabellen 2 und 3 zeigen eine Übersicht über verfügbare Therapeutika.

**TABELLE 2:** Topische Fertigpräparate zur Therapie der kutanen Rosazea bei Kindern und Jugendlichen (Auswahl).

**Fertigpräparat**	**Mögliche Dosierung bei Rosazea im Kindes‐ und Jugendalter**	**Zulassung für Rosazea bei Erwachsenen**	**Zulassung im Kindes‐ und Jugendalter**	**Referenz**
Azelainsäure 15% (Gel)	2 x tgl. über 4–12 Wochen	Ja	Ja (Acne vulgaris)	*44*
Brimonidin 0,3% (Gel)	1 x tgl. bei Bedarf	Ja	Nein	*2*
Erythromycin 2% (Lösung)	2 x tgl. über 4–12 Wochen	Nein	Ja (Acne vulgaris)	*33*
Ivermectin 1% (Creme)	1 x tgl. über 12 Wochen	Ja	Nein	*2* *^,^* *45*
Pimecrolimus 1% (Creme)	1 x tgl. bis zur Besserung	Nein	Ja (atopische Dermatitis)	*46*
Tacrolimus 0,03% oder 0,1% (Salbe)	1 x tgl. bis zur Besserung	Nein	Ja (atopische Dermatitis)	*46*
Metronidazol 0,75% (Gel, Lotion, Creme)	2 x tgl. über 12 Wochen	Ja	Nein	*2* *^,^* *3* *^,^* *30* *^,^* *48*

**TABELLE 3:** Systemtherapeutika der kutanen Rosazea bei Kindern und Jugendlichen (Auswahl).

Präparat	**Mögliche Dosierung im Kindes‐ und Jugendalter**	**Zulassung für Rosazea (bei Erwachsenen)**	**Zulassung im Kindes‐ und Jugendalter**	**Referenz**
Azithromycin	10 mg/kg KG/Tag (an 3 aufeinander folgenden Wochentagen)	Nein	Ja (akute Infektionen)	^48^
Clarithromycin	15 mg/kg KG/Tag als 2 Gaben	Nein	Ja (akute Infektionen)	^32^, ^33^
Erythromycin	30–50 mg/kg KG/Tag	Nein	Ja (akute Infektionen)	^2^, ^7^, ^25^, ^40^, ^50^
Doxycyclin	2,2–4,4 mg/kg KG/d vom 8.–11. Lebensjahr. 40–100 mg/Tag ab dem 12. Lebensjahr	Ja	Ja, ab dem 9. Lebensjahr (akute Infektionen)	^2^, ^7^
Isotretinoin	0,1–0,3 mg/kg KG/Tag	Nein	Ja, ab dem 12. Lebensjahr (Acne vulgaris)	^28^, ^51^, ^52^
Ivermectin	200–250 µg/g als Einzeldosis	Nein	Ja, ab 15 kg (Skabies)	^19^, ^45^

### Topische Therapie

Die topische Anwendung von Metronidazol bei pädiatrischen Patienten, insbesondere bei PPR und POR, hat eine gute Wirksamkeit gezeigt, speziell auch in Kombination mit systemischen Antibiotika.^2^, ^3^ Eine Zulassung von topischem Metronidazol besteht im Kindesalter jedoch nicht. Auch Azelainsäure kann gut bei pädiatrischen Patienten eingesetzt.^44^ Das vorübergehende Hautbrennen zu Therapiebeginn sollte insbesondere bei der Anwendung im Kindesalter eingesetzt werden. Hilfreich kann eine anfänglich reduzierte Anwendungsfrequenz mit einem Einschleichen über jeden zweiten Tag sein. In einer kleinen Kohorte pädiatrischer Patienten mit PPR und POD ergab sich eine gute Wirksamkeit von topischem Ivermectin bei guter Verträglichkeit.^45^ Vorteilhaft ist die einmal tägliche Applikation. Topische Calcineurininhibitioren sind eine weitere Therapieoption, werden gut vertragen und können insbesondere bei Steroid‐induzierter Rosazea erwogen werden.^46^ Weitere topische Präparate sind Erythromycin, Clindamycin, Permethrin und Benzoylperoxid.^47^

### Systemtherapie

Schwere und ausgedehnte Formen einer Rosazea im Kindes‐ und Jugendalter können auch Systemtherapeutika erforderlich machen. Diese werden in der Regel kombiniert mit topischen Maßnahmen über einen Zeitraum von 8–12 Wochen eingesetzt. In der Literatur werden Makrolide, Tetrazykline, Metronidazol, Isotretinoin und Ivermectin erwähnt.^2^, ^45^ Makrolide können bereits vor dem 9. Lebensjahr sicher eingesetzt werden. Azithromycin in einer Dosierung von 10 mg/kg Körpergewicht (KG) über circa 6 Wochen wurde in einer Fallserie von 222 Kindern mit POD mit gutem Therapieansprechen und guter Verträglichkeit angewendet.^48^ Vorteilhaft erscheint hier die intermittierende Einnahme an drei aufeinanderfolgenden Wochentagen. Alternativen stellen Erythromycin und Clarithromycin dar.^2^, ^33^, ^49^ Auch beim IFAG kann eine Therapie mit Makrolid‐Antibiotika erwogen werden.^33^, ^50^ Doxycyclin kann ab dem 9. Lebensjahr bei Kindern mit Rosazea eingesetzt werden.^2^ Bei jüngeren Patienten ist dies aufgrund des Risikos für Zahnschmelz‐ oder Knochendefekte kontraindiziert.

Es existieren nur wenige Fallberichte über Behandlungsversuche mit Isotretinoin bei Kindern mit schwerer Krankheitsausprägung. Eine niedrige dosierte Therapie über circa 6 Monate führte zu einem guten Therapieansprechen.^28^, ^51^ Weiterhin führte eine Therapie mit Isotretinoin zur einer schnellen Remission eines IFAG nach 2 Monaten.^52^ Einzeldosen von Ivermectin bei Kindern und Jugendlichen mit PPR wurde bis auf eine temporäre Schuppung der Haut zu Therapiebeginn gut vertragen und zeigten eine gute Wirksamkeit.^45^

### Therapie der Ophthalmorosacea

Für die Behandlung der OR ist häufig ein kombiniertes langfristiges Behandlungsregime notwendig. Als Basismaßnahmen der OR werden Lidrandhygiene und Tränenersatzmittel empfohlen. Als Topika werden Antibiotika (Azithromycin, Moxifloxacin, Gentamycin), Steroide (Hydrocortison) und Ciclosporin in der Literatur erwähnt. Starosta et al. beschreiben in einer kleinen Fallserie die intermittierende Anwendung von Azithromycin‐Augentropfen 1,5% in Kombination mit Lidrandhygiene.^53^

Unter der Vorstellung, dass *Demodex folliculorum* eine Rolle bei der Ausbildung der Blepharitis spielt, werden lokale und systemische Maßnahmen empfohlen (Teebaumöl‐getränkte Tücher, orales Ivermectin, teilweise kombiniert mit oralem Metronidazol).^9^

Die Systemtherapie erfolgt mit Makroliden (Azithromycin, Erythromycin, Roxithromycin), Tetrazyklinen (Doxycyclin, Minocyclin) oder Metronidazol über mindestens 3–6 Monate.^49^, ^54^ Eine Einzeldosis von Ivermectin führte ebenfalls bei einer 12‐jährigen Patientin mit schwerer PPR mit okulärer Beteiligung zur kompletten Remission.^19^ Eine interdisziplinäre Diagnostik und Therapie durch Dermatologen und Ophthalmologen ist anzustreben.

### Apparative Therapieoptionen

Für die Anwendung apparativer Therapiemaßnahmen wie Laser oder intensiver gepulster Lichtquelle (IPL) liegt bei pädiatrischen Patienten mit Rosazea nur wenig Evidenz vor. Insbesondere bei Jugendlichen erscheint der Einsatz analog zum Erwachsenenalter aber denkbar.^9^

## VERLAUF UND PROGNOSE

Die Mehrheit der pädiatrischen Patienten, die eine temporäre Systemtherapie erhielten, blieben im weiteren Verlauf nach Jahren ohne Rezidiv.^2^, ^33^ Unklar bleibt, ob betroffene Kinder und Jugendliche im Erwachsenalter erneut das Risiko der Entwicklung einer Rosazea aufweisen.^10^ Hierfür sind weitere Studien zur Erfassung der Langzeitverläufe erstrebenswert.

## FAZIT

- Die Diagnosestellung der Rosazea im Kindes‐ und Jugendalter erfolgt auf der Grundlage des klinischen Befundes, der sich meist sehr ähnlich zur Erkrankung im Erwachsenenalter präsentiert.
- Sonderformen im Kindes‐ und Jugendalter sind die periorifizielle Rosazea oder das idiopathische, faziale, aseptische Granulom (IFAG).
- Die Kenntnis des IFAG ist wichtig, um unnötige diagnostische und operative Eingriffe zu vermeiden.
- Kinder und Jugendliche sind häufiger von einer okulären Beteiligung betroffen, diese Form muss erkannt und entsprechend therapiert und überwacht werden. Eine interdisziplinäre Therapie mit Ophthalmologen ist anzustreben.
- Alle Therapiemaßnahmen erfolgen im Off‐Label‐Use.
- Das topische Therapiespektrum umfasst Azelainsäure, Brimonidin, Calcineurininhibitioren, Erythromycin, Ivermectin oder Metronidazol.
- Bei schwerer Krankheitsausprägung kann eine Systemtherapie mit Makrolid‐Antibiotika (Erythromycin, Azithromycin), Metronidazol oder ab dem 9. Lebensjahr mit Doxycyclin erfolgen. Als Alternativen können Ivermectin und Isotretinoin erwogen werden.

## DANKSAGUNG

Open access Veröffentlichung ermöglicht und organisiert durch Projekt DEAL.

## INTERESSENKONFLIKT

A.V. ist Beraterin und Dozentin für Amryt, Bayer Healthcare, Galderma Laboratorium GmbH, Pfizer, Sanofi Regeneron. U.B.‐P. ist Beraterin, Dozentin oder hat klinische Studien durchgeführt für Abbvie, Amryt, Bayer Healthcare, Boots Healthcare, Cantabria Labs, Cassiopeia, CeraVe, Concert Pharmaceuticals, Dermocosmétique Vichy, Galderma Laboratorium GmbH, Eli Lilly, LEO‐Pharma, Novartis, Mayne Pharma, Pfizer, Pierre Fabre, Sanofi Regeneron. U.P. ist Berater für Abbvie, Alcon, Allergan, Alimera, Bausch und Lomb, Bayer, Novartis, Santen, Thea. S.K. und K.S geben keine Interessenkonflikte an.
